# Antioxidant Efficacy of a Standardized Red Orange (*Citrus sinensis* (L.) Osbeck) Extract in Elderly Subjects: A Randomized, Double Blind, Controlled Study

**DOI:** 10.3390/nu14204235

**Published:** 2022-10-11

**Authors:** Vincenzo Nobile, Marta Pisati, Enza Cestone, Violetta Insolia, Vincenzo Zaccaria, Giuseppe Antonio Malfa

**Affiliations:** 1 R&D Department, Complife Italia S.r.l., 27028 San Martino Siccomario, PV, Italy; 2 Alma Mater Europea, 6000 Koper, Slovenia; 3 R&D Department, Bionap S.r.l., 95032 Belpasso, CT, Italy; 4 Department of Drug and Health Science, University of Catania, Viale A. Doria, 95125 Catania, CT, Italy; 5 CERNUT-Research Centre on Nutraceuticals and Health Products, University of Catania, Viale A. Doria, 95125 Catania, CT, Italy

**Keywords:** plant secondary metabolites, oxidative stress, aging, red orange extract, food supplement, menopause

## Abstract

The world population is rapidly aging. This should cause us to reflect on the need to develop a new nutritional approach to mitigate the accumulation of reactive oxygen species (ROS)-induced damage. A randomized, double blind, controlled study was carried out on 60 elderly male and female subjects. Product efficacy was measured before and after 2 and 8 weeks of product intake. The reduced (GSH) and oxidized (GSSG) glutathione concentrations in the erythrocytes and the reactive oxygen metabolites (d-ROMs) hematic concentration were measured to assess the antioxidant efficacy. The tumor necrosis factor-alpha (TNF-α) levels in the serum were measured to assess the anti-inflammatory effectiveness. The wellbeing was assessed by Short Form Health Survey (SF-36) questionnaire (male) and by Menopause Rating Scale (MRS) (female). Blood, urine analysis and electrocardiography (ECG) were carried out to assess the product’s safety. The results showed that GSH/GSSG ratio increased by 22.4% and 89.0% after 2 and 8 weeks of product intake. Serum TNF-α levels decreased by 2.5% after 8 weeks of product intake. The SF-36 QoL and the MRS questionnaire outputs indicate, preliminarily, a positive effect of the extract intake in ameliorating the wellbeing of both male and female subjects. The product was well-tolerated. Our findings suggest that the test product has antioxidant and anti-inflammatory efficacy and has a positive effect on the wellbeing of elderly female and male subjects.

## 1. Introduction

Citrus fruits are a source of high-value bioactive ingredients. Their consumption has been associated with positive effects on human health. Red (or blood) oranges (*Citrus sinensis* (L.) Osbeck) are rich in anthocyanins (95% of which are represented by cyanidin-3-glucoside and cyanidin-3-6″-malonyl-glucoside), flavanones (hesperidin and narirutin), and hydroxycinnamic acids (caffeic acid, coumaric acid, sinapic, and ferulic acid) [1,2]. The antioxidant efficacy of these phenolic compounds is well known and recognized [3,4]. Previous in vivo and in vitro studies have also reported that citrus flavonoids have anti-inflammatory properties [5,6,7], beneficial effects on metabolic syndrome [8,9,10], protect the skin from aging and photoaging [11,12,13,14], can increase neuronal signaling [15], have neuroprotective effects [16,17,18], and cross the blood–brain barrier [19].

Evidence describing the effects of citrus flavonoids on the modulation of aging and aging-related disease is increasing [20,21]. The rationale for studying the effects of dietary intervention stems from evidence involving free radicals in aspects related to the aging process. In fact, it is well-known that age-dependent altered physiological conditions are a cumulative response to alterations induced by reactive oxygen species (ROS) [22]. 

The WHO estimates that between 2015 and 2050, the proportion of the world’s population over 60 years will nearly double from 12% to 22% [23]. This would have a great impact on social and health care expenditures. Developing a nutritional antiaging approach is then of importance in a rapidly aging world population. 

Aging is a complex process that may involve progressive oxidative damage of macromolecules by oxygen radicals leading to progressive loss of functionality. It is well established that the impairment of antioxidant defenses leads to a chronic inflammatory state characterized by an increase in circulating cytokines such as tumor necrosis factor-alpha (TNF-α) and interleukin 6 (IL-6) [24,25]. Various cellular enzymes, or antioxidants from either diet or biosynthesis, are the main weapons for keeping ROS levels within a physiological range [26]. Glutathione (GSH) is one of the most important antioxidants present in all cells; it is synthesized from cysteine, glycine, and glutamate amino acids [27]. Monomeric GSH is involved in a redox reaction by producing its oxidized form glutathione disulfide (GSSG). GSH levels have been reported to decline with old age [28]. Moreover, chronic reduction of the GSH/GSSG ratio in the blood is a marker of elevated oxidative stress [29]. In women, menopause is a normal consequence of aging. It has been proposed that the depletion of estrogen in post-menopause could cause oxidative stress, in addition to the known symptoms [30,31].

The onset of menopause is one of the most critical phases in a woman’s life span and it is associated with a decrease in confidence and self-esteem, influencing the quality of life of menopausal women [32,33]. Hormone replacement therapy (HRT) would be the most intuitive way to combat these changes and represent the first choice in the treatment of menopausal symptoms [34,35,36]; however, the 2002 Women’s Health Initiative (WHI) study showed that hormone replacement therapy increased the risk of breast cancer, stroke, and coronary heart disease in healthy postmenopausal women [37,38]. Nonhormonal therapies are mostly developing, and it is not unusual that women often request a “natural” approach for their menopausal symptoms [39].

In previous in vitro studies on human keratinocytes, the extract showed anti-inflammatory efficacy [2], protection against UVB-induced damage [12], and antioxidant properties [40]. These results were confirmed by in vivo studies on humans in which the extract showed to be effective in reducing the oxidative stress in subjects exposed to air pollutants [11], in protecting the skin from the UVB-induced skin erythema [13,14], and in decreasing the photoaging clinical signs [14,41]. This study investigated the efficacy of a standardized Red Orange Complex extract (ROC^TM^, Bionap Srl, 95032 Piano Tavola Belpasso, CT, Italy) on elderly subjects, exploring more in detail the antioxidant efficacy and, preliminarily, the effect of the extract on the wellbeing and on the menopause symptoms of both male and female subjects, respectively.

## 2. Materials and Methods

### 2.1. Study Design Description

The design of the study was as follows: single-center, stratified (balanced randomization [1:1] of both male and female subjects), randomized, double-blind, placebo-controlled study conducted in Milan (Italy). 

All the study procedures were conducted in accordance with the World Medical Association’s (WMA) Helsinki Declaration and its amendments. The study protocol and the informed consent form were approved by the “Comitato Etico di Ateneo (CEA) Università della Calabria” (ref. no. 0023482 by 27.05.2021). The trial was registered at ISRCTN registry, number ISRCTN11550896, https://doi.org/10.1186/ISRCTN11550896 (accessed on 12 September 2022).

The informed consent was obtained for all the subjects participating in the study before the start of the study.

### 2.2. Eligibility Criteria for Participants

Eligible subjects were all male (50%) and female (50%) healthy adults aged between 45 and 60 years old (extremes included). Exclusion criteria were chosen to minimize confounding factors. A list of both the inclusion and the exclusion criteria are reported in the Supplementary Table S1.

### 2.3. Settings and Locations

Subjects were enrolled at Complife Italia Srl San Martino Siccomario (Pavia, Italy) facility. Complife is an independent international group of testing international laboratories specialized in the *in vitro* and *in vivo* safety and efficacy assessment of cosmetics, food supplements and medical devices.

### 2.4. Intervention

The active test item was a food supplement containing a standardized Red Orange Complex extract (ROC^TM^, Bionap Srl, 95032 Piano Tavola Belpasso, CT, Italy) obtained from 3 different pigmented, red, Sicilian oranges (*Citrus sinensis*) varieties (Moro, Tarocco, and Sanguinello). The extract contained (*w/w*): 1.8–2.2% hydroxycinnamic acids, 2.8–3.2% anthocyanins (cyanidin-3-glucoside), 5.5–6.5% ascorbic acid, and 8.5–9.5% flavanones (hesperidin, narirutin). The composition (per capsule) of the active food supplement was as follows: 100 mg Red Orange Complex 200 mg Maltodextrin, 108 mg capsule jelly size 0.2 mg titanium dioxide. The placebo product contained (per capsule) 300 mg Maltodextrin, 108 mg capsule jelly size 0.2 mg titanium dioxide. The posology for both the active and the placebo products was 1 capsule a day after breakfast.

### 2.5. Randomization and Masking

Half of the participants were then randomly assigned to receive the active or the placebo product. A restricted randomization list was created by dr. Vincenzo Nobile (VN) using PASS 11 (version 11.0.8; PASS, LLC. Kaysville, UT, USA) statistical software running on Windows Server 2008 R2 Standard SP1 64-bit edition (Microsoft, Redmond, WA, USA). The randomization sequence was stratified with 1:1 allocation using the “Efron’s biased coin” algorithm. The allocation sequence was concealed by the study director (VN) in sequentially numbered, opaque, and sealed envelopes. The unblinded randomization sequence was folded to render the envelope impermeable to intense light, sealed and stored in a safe place. A masked allocation sequence was prepared by the study director (VN). This allocation sequence was used by the staff delivering the intervention. The study adhered to established procedures to maintain separation between the investigator and its collaborators and the staff that delivered the intervention. The investigator and its collaborators who obtained outcome measurements were not informed of the (masked) product group assignment. The staff who delivered the intervention did not take outcome measurements. Subjects, investigators, and collaborators were kept masked to products assignment.

### 2.6. Primary and Secondary Objectives and Outcome Measures

The primary objective was the assessment of the efficacy of the product in improving the systemic antioxidants pool after 2 and 8 weeks of product use. The primary outcome measure was the concentration of glutathione in erythrocytes and the d-ROMS hematic concentration.

The secondary objective was the assessment of the anti-inflammatory activity, the efficacy in improving the wellbeing in male subjects and the menopausal symptoms in female subjects. The study further assessed the safety of use of the test product. The secondary outcome measures were serum TNF-a levels, wellbeing by SF-36 QoL questionnaire, and menopause symptoms by Menopause Rating Scale. 

The study flow and the schedule of assessment chart can be found in Supplementary Figure S1.

#### 2.6.1. Concentration of Glutathione in Erythrocytes

Measurement of erythrocyte reduced (GSH) and oxidized (GSSG) glutathione was performed using the GSH+GSSG/GSH Assay Kit (catalog no. ab239709) from Abcam (Discovery Drive, Cambridge Biomedical Campus, Cambridge, CB2 0AX, UK), according to the manufactures’ instructions. Briefly, blood samples were collected into tubes containing anticoagulants and centrifuged at 1000× *g* for 10 min at 4 °C, the supernatant and the white buffy layer were discarded, erythrocytes were lysed with 4 vol of glutathione buffer for 10 min on ice, 1 vol of 5% 5-Sulfosalicylic acid (SSA) were added, mixed well, and centrifuged at 8000× *g* for 10, samples were then stored at −20 °C until further analysis.

#### 2.6.2. d-ROMs Hematic Concentration

The hematic concentration of the Reactive Oxygen Metabolites (d-ROMs) was measured using FRAS 5 device (H&D srl, Parma, Italy). The d-ROMs fast test by FRAS 5 allows us to perform the d-ROMs (fast) test directly on capillary blood (finger-prick). The use of FRAS 5 in measuring d-ROMS levels is supported by over 700 scientific references. The d-ROMS fast test references values are as follows: 250–300 U.CARR → Normal value, 301–320 U.CARR → Borderline, 321–340 U.CARR → Low level of oxidative stress, 341–400 U.CARR → Middle level of oxidative stress, 401–500 U.CARR → High level of oxidative stress, >500 U.CARR → Very high level of oxidative stress.

#### 2.6.3. Serum TNF-α Levels

Measurement of TNF-α levels in serum was performed using the Human TNF alpha ELISA Kit (catalog no. ab46087) from Abcam (Discovery Drive, Cambridge Biomedical Campus, Cambridge, CB2 0AX, UK), according to the manufactures’ instructions. Briefly, blood samples were centrifuged at 2000× *g* for 10 min and the collected serum was stored at −20 °C until further analysis. 

#### 2.6.4. Short Form Health Survey (SF-36) Questionnaire

The SF-36 questionnaire consists of 36 items, which were used to calculate eight subscales: physical functioning, role limitations due to physical health, role limitations due to emotional problems, energy/fatigue, emotional well-being, social functioning, pain, and general health. Scores for the SF-36 scales range between 0 and 100 (Supplementary Table S2), with higher scores indicating a better QoL.

#### 2.6.5. Menopause Rating Scale (MRS)

The MRS was developed and validated to evaluate the severity of menopause-related complaints [22]. A 5-point rating scale from zero (no complaint) to four (extremely severe symptoms) permits to describe the severity of complaints of each item. The MRS consisted of eleven questions (Supplementary Table S3).

#### 2.6.6. Blood and Urine Analysis

Blood and urine analysis were performed according to the current protocols of accredited clinical analysis laboratories. The following parameters were analyzed before and after 8 weeks of product use:

1. Complete blood cell count: White Blood Cells (WBC), Red Blood cells (RBC), Haemoglobin (Hb), Haematocrit (Hct), and Platelet count (PLT);
2. Biochemistry test: Blood Urea Nitrogen (BUN, azotemia), Cholesterol, High-Density Lipoprotein Cholesterol (HDL-C), Low-Density Lipoprotein Cholesterol (LDL-C), Triglycerides, Albumin, Total bilirubin, Alkaline Phosphatase (ALP), Gamma-glutamyl Transferase (r-GT), Creatinine, High-sensitivity C-reactive Protein (hs-CRP);
3. Urinalysis: Specific Gravity, pH, White Blood Cells (leukocytes, WBC), Occult blood (erythrocytes), Nitrite, Protein, Glucose, Ketone body, Urobilinogen, Bilirubin.

### 2.7. Statistical Analysis

We used a two-way Student’s *t*-test for parametric data, while a Wilcoxon (intragroup analysis) or Mann–Whitney test (intergroup analysis) was used for non-parametric data. Before any statistical analysis took place the normal distribution of each dataset was checked by Shapiro–Wilk W test. The statistical analysis was carried out using NCSS 10 (version 10.0.7 for Windows; NCSS, Kaysville, UT, USA) running on Windows Server 2008 R2 Standard SP1 64-bit edition (Microsoft, WA, USA). A *p* < 0.05 was considered statistically significant. The level of significant was reported as follows: * *p* < 0.05, ** *p* < 0.01, and *** *p* < 0.001.

## 3. Results

### 3.1. Participants and Product Tolerability

**Figure 1:**
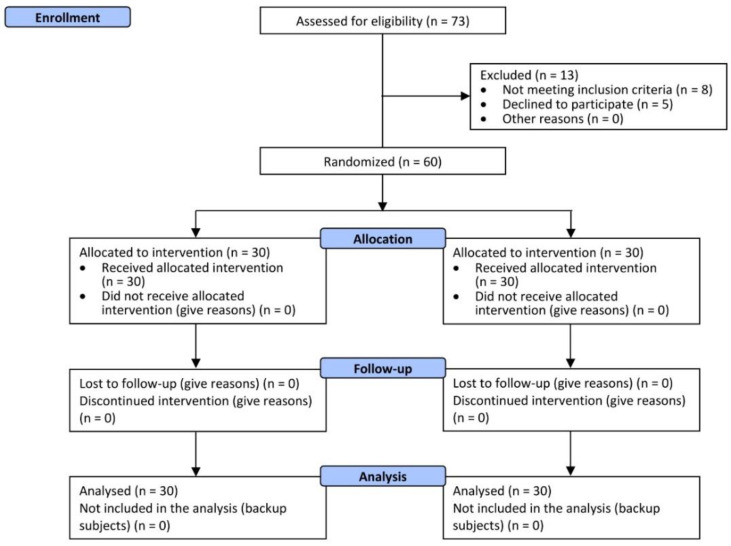
Participants flow diagram.

A total of 60 male and female subjects were successfully randomized. Thirty (n = 30) subjects were allocated to each treatment arm (Figure 1). The population was male and female (1:1 ratio, n = 15 per treatment arm). Demographic and baseline characteristics (Supplementary Table S4) were similar across treatment arms, indicating unbiased randomization and the absence of covariates. No drop-outs were recorded. All subjects were included in the efficacy and safety analysis data set. All the tested products were well tolerated. No adverse reactions occurred during the study period. The absence of adverse events was also confirmed sub-clinically by the blood and urine tests (Supplementary Tables S5 and S6).

### 3.2. Primary Endpoints: The Systemic Antioxidants Pool

**Figure 2:**
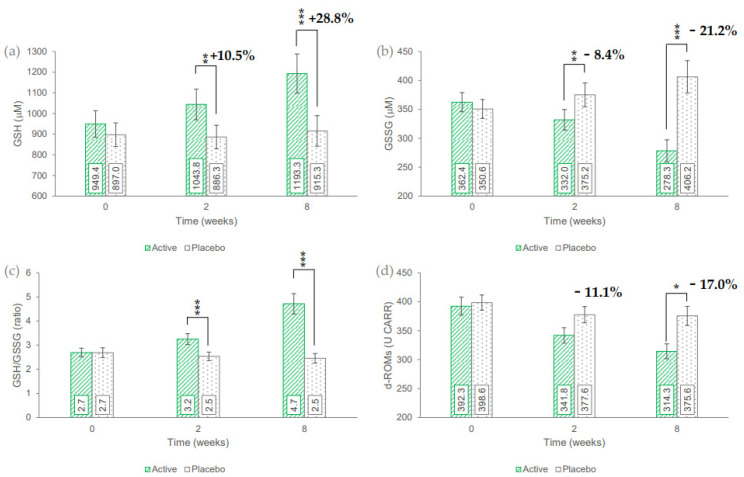
(**a**) Reduced glutathione (GSH). (**b**) Oxidized glutathione (GSSG). (**c**) GSH/GSSG ratio. (**d**) d-ROMs hematic concentration. Data are average (± standard error). The intergroup statistical analysis is reported above the bar as follows: * *p* < 0.05, ** *p* < 0.01, and *** *p* < 0.001.

The primary endpoints related to efficacy were measured before and after 2 and 8 weeks of product intake. Data are reported in Figure 2.

In the active treatment arm, the basal (949.4 ± 63.9 mM) concentration of GSH in erythrocytes was statistically significant increased by 10.5% (1043.8 ± 74.4 mM, *p* = 0.0021) and 28.8% (1193.3 ± 95.0 mM, *p* = 0.0007) after 2 and 8 weeks of use, respectively, while do not was statistically significant in the placebo treatment arm. The GSH variation vs. baseline in the active treatment arm was statistically significant when compared to the variation in the placebo treatment arm (*p* = 0.0030 and *p* = 0.0005, after 2 and 8 weeks, respectively).

In the active treatment arm, the basal (362.4 ± 16.7 mM) concentration of GSSG in erythrocytes was statistically significant decreased by 8.4% (332.0 ± 17.8 mM, *p* = 0.0021) and +21.2% (278.3 ± 19.0 mM, *p* = 0.0001) after 2 and 8 weeks of use, respectively. The GSSG variation in the placebo treatment arm was not statistically significant. The GSSG variation vs. baseline in the active treatment arm was statistically significant when compared to the variation in the placebo treatment arm (*p* = 0.0012 and *p* = 0.0001, after 2 and 8 weeks, respectively).

Similar consideration can be drawn for the GSH/GSSG ratio that increased by 22.4% and 89.0% after 2 and 8 weeks of use, respectively.

In the active treatment arm, the basal (392.3 ± 15.5 U.CARR) levels of d-ROMs were statistically significant decreased by 11.1% (341.8 ± 13.4 U.CARR, *p* = 0.0007) and 17.0% (314.3 ± 12.9 U.CARR, *p* = 0.0001) after 2 and 8 weeks of use, respectively, while do not was statistically significant in the placebo treatment arm. The d-ROMs variation vs. baseline in the active treatment arm was statistically significant when compared to the variation in the placebo treatment arm (*p* = 0.0207, after 8 weeks, respectively).

### 3.3. Secondary Endpoints

The secondary endpoints related to efficacy were measured before and after 2 and 8 weeks of product intake. The following parameters were measured: Serum TNF-α levels, SF-36 QoL questionnaire, MRS questionnaire. 

The wellbeing of the male subjects, in the active treatment arm, seemed to be improved in the role limitation (both physical health and emotional problems), energy/fatigue, emotional well-being, and general health domains (Table 1). The variation vs. baseline in these domains was statistically significant vs. baseline. Some differences between active and placebo were borderline statistically significant. The obtained data suggest a positive effect of the test product in improving the QoL even if more data are needed to confirm or to improve the robustness of the obtained data. 

**Figure 3:**
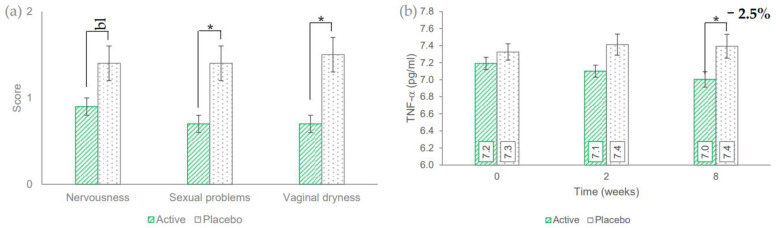
(**a**) Output of selected items of Menopause rating scale questionnaire 8 weeks after product use. (**b**) Serum TNF-α levels. Data are average (± standard error). The intergroup statistical analysis is reported above the bar as follows: bl borderline, * *p* < 0.05.

The wellbeing of the menopausal women, in the active treatment arm, was statistically significant improved for all the MRS questionnaire items (Table 2). Some items were statistically significant in both intragroup and intergroup analysis (item 5 at T8 *p* = 0.0623, item 8 at T8 *p* = 0.0466, and item 10 at T8 *p* = 0.0115, Figure 3a). The obtained data suggest a positive effect of the test product in improving menopausal symptoms even if more data are needed to confirm or to improve the robustness of the obtained results. 

In the active treatment arm, the basal (7.19 ± 0.07 pg/mL) concentration of TNF-a was statistically significant decreased by 2.5% (7.00 ± 0.09 pg/mL, *p* = 0.0335) after 8 weeks of use, respectively (Figure 3b), while do not was statistically significant in the placebo treatment arm. After 8 weeks of product use, the TNF-a variation vs. baseline in the active treatment arm was statistically significant (*p* = 0.0420) when compared to the variation in the placebo treatment arm.

## 4. Discussion

Aging and age-dependent altered conditions are a cumulative response to alterations induced by ROS [22]. 

Since the world population is rapidly aging [23] a nutritional approach to mitigate the effects of aging would be of importance in the next years. Eating well could be the best way to mitigate the age-related conditions. This is the new awareness of the role of nutrition in skin health and specific dietary components have emerged as an effective alternative strategy to prevent and mitigate the symptoms of aging. In this view natural phenolic compounds have been shown to play an important role in health benefits because of their high antioxidant capacity [3,4]. Another advantage of the naturally derived ingredients is their acceptance by Consumers, that see this category of ingredients as safe, non-toxic, and environmentally friendly [42].

In this study, we investigated the efficacy of a standardized red orange (*Citrus sinensis* (L.) Osbeck) extract obtained from the juice of three pigmented varieties of Sicilian blood orange (Moro, Tarocco and Sanguinello) that grown in a particular area surrounding Europe’s most active volcano, Mt. Etna (Catania, CT, Italy). This extract contains anthocyanins (cyanidin-3-glucoside), hydroxycinnamic acids, flavanones (hesperidin, narirutin), and ascorbic acid.

The antioxidant properties of the test product were demonstrated in previous in vitro and in vivo studies [11,14,32]. This study confirmed the well-known antioxidant efficacy of *Citrus sinensis*. The product was, in fact, effective in increasing the GSH hematic level while decreasing GSSG levels A similar trend was observed by the results of the d-ROMs test in serum, where the level of oxidative stress went from the scoring “oxidative stress” to the scoring “borderline level of oxidative stress” and “normal value” after 2 and 8 weeks of product use, respectively.

In a previous in vitro study, the test product showed an effect in modulating the production of ICAM-1, MCP-1 and IL-8 in normal keratinocytes NCTC 2544 stimulated with IFN-a and histamine [2]. In line with that finding, this study demonstrated a decrease of systemic TNF-a levels in serum.

Interesting, even if preliminary, results were obtained during the assessment of the wellbeing in men (by SF-36 QoL questionnaire) and the rating of menopausal symptoms in women (by MRS questionnaire). Male subjects seemed to be improved in the role limitation (both physical health and emotional problems), energy/fatigue, emotional well-being, and general health domains while female subjects showed an improvement in the basal scoring of all the eleven items of the MRS questionnaire. The best results were observed in the reduction of the items related to “nervousness”, “sexual problems”, and “vaginal dryness”. The improvement of these parameters was significant not only compared to the baseline but also compared to the placebo treatment arm. These findings should be further investigated in future studies. 

The limitations of the study concern the relatively low number of subjects and its limiting effect on the intergroup statistics of subjective parameters (SF-36 QoL and MRS questionnaire).

## 5. Conclusions

The intake of 100 mg/day of the standardized red orange (*Citrus sinensis* (L.) Osbeck) extract containing anthocyanins, hydroxycinnamic acids, flavanones, and ascorbic acid was effective in improving the systematic oxidative stress and in decreasing the levels of the pro-inflammatory cytokine TNF-a. Interestingly the extract demonstrated for the first time to have a positive effect on the wellbeing of both men and women. In women, the reduction of the items related to menopause such as “nervousness”, “sexual problems”, and “vaginal dryness” are important findings to be considered in supportive treatments for elderly females. However, this data needs to be confirmed and further investigated in a larger group of subjects.

## Figures and Tables

**Table 1:** SF-36 QoL questionnaire output. The intragroup statistical analysis is reported next each value (apex). Legend T0 baseline (first day of study), T2 follow-up visit after 2 weeks of product use, T8 follow-up visit after 8 weeks of product use. ‡ Statistically (borderline 0.06 < *p* < 0.08) significant vs. placebo.

	Active			Placebo		
	T0	T2	T8	T0	T2	T8
1. Physical functioning	85.7	84.5	88.3	83.0	81.3	89.8 *^p^* ^= 0.0143^
2. Role limit. (physical health)	64.2	76.7 *^p^* ^= 0.0211^	83.3 *^p^* ^= 0.0021, ‡^	69.2	78.3	70.0
3. Role limit. (emot. probl.)	54.4	78.9 *^p^* ^= 0.0015^	78.9 *^p^* ^= 0.0190^	65.6	68.9	78.9 *^p^* ^= 0.0498^
4. Energy/fatigue	59.5	62.7 *^p^* ^= 0.0522^	63.3	61.7	61.5	63.7
5. Emotional well-being	35.3	31.3 *^p^* ^= 0.0455^	28.3 *^p^* ^= 0.0136^	30.7	32.5	26.1 *^p^* ^= 0.0404^
6. Social functioning	53.8	53.3	54.2 ^‡^	59.6	58.8	60.0
7. Pain	68.5	71.3	74.2	74.5	72.9	75.9
8. General health	45.8	49.8 *^p^* ^= 0.0010^	52.7 *^p^* ^= 0.0030^	46.3	48.5	50.3 *^p^* ^= 0.0874^
9. Physical functioning	85.7	84.5	88.3	83.0	81.3	89.8 *^p^* ^= 0.0143^

**Table 2:** Menopause Rating Scale (MRS) questionnaire output. The intragroup statistical analysis is reported as follows: * *p* < 0.05, ** *p* < 0.01, and *** *p* < 0.001. Legend T0 baseline (first day of study), T2 follow-up visit after 2 weeks of product use, T8 follow-up visit after 8 weeks of product use. ‡ Statistically significant vs. placebo.

	Active			Placebo		
	T0	T2	T8	T0	T2	T8
1. Hot flashes and sweating	2.6	1.9 *^p^* ^= 0.0052^	1.7 *^p^* ^= 0.0033^	2.1	1.9	1.6
2. Heart problems	1.7	1.4	0.9 *^p^* ^= 0.0230^	1.8	1.5	1.4
3. Sleep problems	2.2	1.5 ^*p* = 0.0292^	1.3 *^p^* ^= 0.0033^	2.1	1.7 *^p^* ^= 0.0339^	1.8
4. Feeling unhappy	1.8	1.5	1.1 *^p^* ^= 0.0261^	1.7	1.7	1.6
5. Nervousness	1.9	1.5	1.0 ^*p* = 0.0020, ‡^	1.9	1.7	1.6
6. Anxiety	1.7	1.3 *^p^* ^= 0.0559^	1.1 *^p^* ^= 0.0087^	1.8	1.7	1.5
7. Phys. and mental fatigue	2.3	1.7 *^p^* ^= 0.0085^	1.3 *^p^* ^= 0.0026^	2.2	1.9	1.9
8. Sexual problems	1.9	1.2 *^p^* ^= 0.0110^	0.7 ^*p* = 0.0010,^^‡^	1.4	1.3	1.4
9. Urinary problems	1.6	1.3	1.0 *^p^* ^= 0.0290^	1.6	1.4	1.5
10. Vaginal dryness	1.8	1.0 *^p^* ^= 0.0163^	0.7 ^*p* = 0.0009,^^‡^	1.8	1.4	1.5
11. Joint and muscle problems	2.0	1.7	1.5 *^p^* ^= 0.0310^	1.8	1.7	1.7

## Data Availability

The data presented in this study are available on request from the corresponding author. The data are not publicly available since they are the property of the sponsor of the study (Bionap Srl, 95032 Piano Tavola Belpasso, CT, Italy).

## Supplementary Materials

The following supporting information can be downloaded at: https://www.mdpi.com/article/10.3390/nu14204235/s1, Table S1: Inclusion and exclusion criteria; Figure S1: Study flow and schedule of assessments chart; Table S2: SF-36 QoL questionnaire; Table S3: Menopause Rating Scale (MRS); Table S4: Subjects’ baseline demographic and clinical characteristics; Table S5: Blood and Urine analysis in the active treatment arm; Table S6: Blood and Urine analysis in the placebo treatment arm.

## References

[1] Chiechio S., Zammataro M., Barresi M., Amenta M., Ballistreri G., Fabroni S., Rapisarda P. (2021). A Standardized Extract Prepared from Red Orange and Lemon Wastes Blocks High-Fat Diet-Induced Hyperglycemia and Hyperlipidemia in Mice. Molecules.

[2] Cardile V., Frasca G., Rizza L., Rapisarda P., Bonina F. (2010). Antiinflammatory Effects of a Red Orange Extract in Human Keratinocytes Treated with Interferon-Gamma and Histamine. Phytother. Res..

[3] Legua P., Modica G., Porras I., Conesa A., Continella A. (2022). Bioactive Compounds, Antioxidant Activity and Fruit Quality Evaluation of Eleven Blood Orange Cultivars. J. Sci. Food Agric..

[4] Saija A., Tomaino A., Lo Cascio R., Rapisarda P., Dederen J.C. (1998). In Vitro Antioxidant Activity and in Vivo Photoprotective Effect of a Red Orange Extract. Int. J. Cosmet. Sci..

[5] Parhiz H., Roohbakhsh A., Soltani F., Rezaee R., Iranshahi M. (2015). Antioxidant and Anti-Inflammatory Properties of the Citrus Flavonoids Hesperidin and Hesperetin: An Updated Review of Their Molecular Mechanisms and Experimental Models. Phytother. Res..

[6] Alam M.A., Subhan N., Rahman M.M., Uddin S.J., Reza H.M., Sarker S.D. (2014). Effect of Citrus Flavonoids, Naringin and Naringenin, on Metabolic Syndrome and Their Mechanisms of Action. Adv. Nutr..

[7] Frasca G., Panico A.M., Bonina F., Messina R., Rizza L., Musumeci G., Rapisarda P., Cardile V. (2010). Involvement of Inducible Nitric Oxide Synthase and Cyclooxygenase-2 in the Anti-Inflammatory Effects of a Red Orange Extract in Human Chondrocytes. Nat. Prod. Res..

[8] Mulvihill E.E., Huff M.W. (2012). Protection from Metabolic Dysregulation, Obesity, and Atherosclerosis by Citrus Flavonoids: Activation of Hepatic PGC1α-Mediated Fatty Acid Oxidation. PPAR Res..

[9] Mahmoud A.M., Hernández Bautista R.J., Sandhu M.A., Hussein O.E. (2019). Beneficial Effects of Citrus Flavonoids on Cardiovascular and Metabolic Health. Oxid. Med. Cell. Longev..

[10] Cardile V., Graziano A.C.E., Venditti A. (2015). Clinical Evaluation of Moro (*Citrus Sinensis* (L.) Osbeck) Orange Juice Supplementation for the Weight Management. Nat. Prod. Res..

[11] Bonina F.P., Puglia C., Frasca G., Cimino F., Trombetta D., Tringali G., Roccazzello A., Insiriello E., Rapisarda P., Saija A. (2008). Protective Effects of a Standardised Red Orange Extract on Air Pollution-Induced Oxidative Damage in Traffic Police Officers. Nat. Prod. Res..

[12] Cimino F., Cristani M., Saija A., Bonina F.P., Virgili F. (2007). Protective Effects of a Red Orange Extract on UVB-Induced Damage in Human Keratinocytes. Biofactors.

[13] Puglia C., Offerta A., Saija A., Trombetta D., Venera C. (2014). Protective Effect of Red Orange Extract Supplementation against UV-Induced Skin Damages: Photoaging and Solar Lentigines. J. Cosmet. Dermatol..

[14] Nobile V., Burioli A., Yu S., Zhifeng S., Cestone E., Insolia V., Zaccaria V., Malfa G.A. (2022). Photoprotective and Antiaging Effects of a Standardized Red Orange (*Citrus Sinensis* (L.) Osbeck) Extract in Asian and Caucasian Subjects: A Randomized, Double-Blind, Controlled Study. Nutrients.

[15] Spencer J.P.E. (2007). The Interactions of Flavonoids within Neuronal Signalling Pathways. Genes Nutr..

[16] Muhammad T., Ikram M., Ullah R., Rehman S.U., Kim M.O. (2019). Hesperetin, a Citrus Flavonoid, Attenuates LPS-Induced Neuroinflammation, Apoptosis and Memory Impairments by Modulating TLR4/NF-ΚB Signaling. Nutrients.

[17] Huang H., Hu C., Xu L., Zhu X., Zhao L., Min J. (2020). The Effects of Hesperidin on Neuronal Apoptosis and Cognitive Impairment in the Sevoflurane Anesthetized Rat Are Mediated Through the PI3/Akt/PTEN and Nuclear Factor-ΚB (NF-ΚB) Signaling Pathways. Med. Sci. Monit..

[18] Zhang S., Tomata Y., Sugiyama K., Sugawara Y., Tsuji I. (2017). Citrus Consumption and Incident Dementia in Elderly Japanese: The Ohsaki Cohort 2006 Study. Br. J. Nutr..

[19] Youdim K.A., Shukitt-Hale B., Joseph J.A. (2004). Flavonoids and the Brain: Interactions at the Blood-Brain Barrier and Their Physiological Effects on the Central Nervous System. Free Radic. Biol. Med..

[20] Ge Y., Chen H., Wang J., Liu G., Cui S.W., Kang J., Jiang Y., Wang H. (2021). Naringenin Prolongs Lifespan and Delays Aging Mediated by IIS and MAPK in Caenorhabditis Elegans. Food Funct..

[21] Yang X., Wang H., Li T., Chen L., Zheng B., Liu R.H. (2020). Nobiletin Delays Aging and Enhances Stress Resistance of Caenorhabditis Elegans. Int. J. Mol. Sci..

[22] Davalli P., Mitic T., Caporali A., Lauriola A., D’Arca D. (2016). ROS, Cell Senescence, and Novel Molecular Mechanisms in Aging and Age-Related Diseases. Oxid. Med. Cell Longev..

[23] Ageing and Health. https://www.who.int/news-room/fact-sheets/detail/ageing-and-health.

[24] Krabbe K.S., Pedersen M., Bruunsgaard H. (2004). Inflammatory Mediators in the Elderly. Exp. Gerontol..

[25] Beyer I., Mets T., Bautmans I. (2012). Chronic Low-Grade Inflammation and Age-Related Sarcopenia. Curr. Opin. Clin. Nutr. Metab. Care.

[26] Sies H. (1997). Oxidative Stress: Oxidants and Antioxidants. Exp. Physiol..

[27] Forman H.J., Zhang H., Rinna A. (2009). Glutathione: Overview of Its Protective Roles, Measurement, and Biosynthesis. Mol. Asp. Med..

[28] Sekhar R.V., Patel S.G., Guthikonda A.P., Reid M., Balasubramanyam A., Taffet G.E., Jahoor F. (2011). Deficient Synthesis of Glutathione Underlies Oxidative Stress in Aging and Can Be Corrected by Dietary Cysteine and Glycine Supplementation. Am. J. Clin. Nutr..

[29] Cysteine Disulfides (Cys-ss-X) as Sensitive Plasma Biomarkers of Oxidative Stress—PubMed. https://pubmed.ncbi.nlm.nih.gov/30643157/.

[30] Rao P.M., Kelly D.M., Jones T.H. (2013). Testosterone and Insulin Resistance in the Metabolic Syndrome and T2DM in Men. Nat. Rev. Endocrinol..

[31] Doshi S.B., Agarwal A. (2013). The Role of Oxidative Stress in Menopause. J. Midlife Health.

[32] Marjoribanks J., Farquhar C., Roberts H., Lethaby A., Lee J. (2017). Long-Term Hormone Therapy for Perimenopausal and Postmenopausal Women. Cochrane Database Syst. Rev..

[33] Gurney E.P., Nachtigall M.J., Nachtigall L.E., Naftolin F. (2014). The Women’s Health Initiative Trial and Related Studies: 10 Years Later: A Clinician’s View. J. Steroid Biochem. Mol. Biol..

[34] Hill D.A., Crider M., Hill S.R. (2016). Hormone Therapy and Other Treatments for Symptoms of Menopause. Am. Fam. Physician.

[35] The NAMS 2017 Hormone Therapy Position Statement Advisory Panel (2017). The 2017 Hormone Therapy Position Statement of The North American Menopause Society. Menopause.

[36] Manson J.E., Chlebowski R.T., Stefanick M.L., Aragaki A.K., Rossouw J.E., Prentice R.L., Anderson G., Howard B.V., Thomson C.A., LaCroix A.Z. (2013). Menopausal Hormone Therapy and Health Outcomes during the Intervention and Extended Poststopping Phases of the Women’s Health Initiative Randomized Trials. JAMA.

[37] Krebs E.E., Ensrud K.E., MacDonald R., Wilt T.J. (2004). Phytoestrogens for Treatment of Menopausal Symptoms: A Systematic Review. Obstet. Gynecol..

[38] Chen L.-R., Ko N.-Y., Chen K.-H. (2019). Isoflavone Supplements for Menopausal Women: A Systematic Review. Nutrients.

[39] De Franciscis P., Colacurci N., Riemma G., Conte A., Pittana E., Guida M., Schiattarella A. (2019). A Nutraceutical Approach to Menopausal Complaints. Medicina.

[40] Morini F., Dusatti F., Bonina F.P., Saija A., Ferro M. (2000). Iron-Induced Lipid Peroxidation in Human Skin-Derived Cell Lines: Protection by a Red Orange Extract. Altern. Lab. Anim..

[41] Tomasello B., Malfa G.A., Acquaviva R., La Mantia A., Di Giacomo C. (2022). Phytocomplex of a Standardized Extract from Red Orange (*Citrus Sinensis*, L. Osbeck) against Photoaging. Cells.

[42] Sansone F., Mencherini T., Picerno P., Lauro M.R., Cerrato M., Aquino R.P. (2019). Development of Health Products from Natural Sources. Curr. Med. Chem..

