# Supplementary material for: Antioxidant Efficacy of a Standardized Red Orange (Citrus sinensis (L.) Osbeck) Extract in Elderly Subjects: A Randomized, Double Blind, Controlled Study
Source: Nutrients. 2022 Oct 11;14(20):4235. doi: 10.3390/nu14204235 (PMC9611767; doi:10.3390/nu14204235)
Supplement: Supplementary file 1 [file nutrients-14-04235-s001.zip › nutrients-1941267-supplementary.pdf]

SUPPLEMENTARY INFORMATION

Antioxidant Efficacy of a Standardized Red Orange (Citrus sinensis (L.) Osbeck) Extract in elderly subjects: A Random-ized, Double Blind, Controlled Study.

Vincenzo Nobile, Marta Pisati, Enza Cestone, Violetta Insolia, Vincenzo Zaccaria and Giuseppe Antonio Malfa.

Supplementary Table S1. Inclusion and exclusion criteria.

No.	Inclusion criteria
01	Men (50%) and women (50%) aged 45-60 years old
02	Subjects with menopausal symptoms (only for women)
03	Subjects naïve to treatment and food supplement that can interfere with the study treatment for the previous 1 month
04	Promise to not use during all the study period topic products/food supplements with similar effect to that one of the products to be tested (antioxidant)
05	Subjects registered with health social security or health social insurance
06	Subjects reading, understanding and signed approval of the informative consent
07	No vegetarian
08	Subjects who will continue, expect reserve, their normal lifestyle
09	Healthy subjects without clinical illness diagnosed with relevant effect on the gastrointestinal system or the motility visceral
10	No pregnant women (only for women)
11	No smokers
12	Subjects available and willing to follow the procedure of the study protocol
13	Subjects able to understand the language used in the investigation centre and the information given
**No.**	**Exclusion criteria**
01	Subjects who do not fit the inclusion criteria
02	Subjects <45 and >60 years old
03	Clinical history with relevant presence of any disorder or administration of drugs/food supplement that can potentially interfere with the treatment under study
04	Lack of compliance defined as not using the correct dose or placebo for >1 week, and inability to give informed consent
05	Subjects who have changed their diet significantly or have been placed on weight reduction products
06	Smokers, obese subjects
07	Subjects changing the eating habits within the 2 weeks previous the screening
08	Pregnancy or subjects planning a pregnancy (only for women)
09	Breastfeeding (only for women)
10	Subjects with a history of drug, alcohol, and other substance abuse
11	Subjects planning to change their lifestyle or physical activity
12	Known food intolerance or food allergy
13	Subjects involved in a clinical or food study within the previous month
14	Subjects who have unstable medical diseases (cardiac arrhythmias or ischemia, uncontrolled hypertension and hypotension, diabetes mellitus, kidney failure)
15	Subjects with a history of paralysis or cerebral vascular accident
16	Subjects with active cancers or on chemotherapy
17	Subjects who have been under diuretics for the previous one months
18	Other factors that limit their ability to cooperate during the study
19	Subjects deprived of freedom by administrative or legal decision or under guardianship
20	Subjects not able to be contacted in case of emergency
21	Subjects planning a hospitalisation during the study

DAYS (D)	T0	T2	T8
Subject information and delivery of the information sheet	✓		
Signature of the informed consent form and photograph authorisation signature	✓		
Demographics data	✓		
Verification of inclusion and non-inclusion criteria	✓		
Randomisation	✓		
Dispensing of daily log (alimentary diary) and investigational products	✓		
Efficacy parameters			
Glutathione level	✓	✓	✓
D-rom test	✓	✓	✓
TNF-α	✓	✓	✓
SF-36 Quality-of-Life Scale	✓	✓	✓
Menopause rating scale (only for women)	✓	✓	✓
Safety parameters			
Complete blood cell count:			
WBC	✓		✓
RBC	✓		✓
Hb	✓		✓
Hct	✓		✓
PLT	✓		✓
Biochemistry test:			
BUN	✓		✓
Cholesterol	✓		✓
Albumin	✓		✓
Total bilirubin	✓		✓
ALP	✓		✓
r-GT	✓		✓
Creatinine	✓		✓
Triglycerides	✓		✓
HDL-C	✓		✓
LDL-C	✓		✓
hs-CRP	✓		✓
Urinalysis:			
Specific Gravity	✓		✓
pH	✓		✓
Wbc	✓		✓
Nitrite	✓		✓
Protein	✓		✓
Glucose	✓		✓
Ketone body	✓		✓
Urobilinogen	✓		✓
Bilirubin	✓		✓
Occult blood (erythrocytes)	✓		✓
ECG (electrocardiography)	✓		✓
Dispensing investigational products	✓		
Dispensing questionnaires (SF-36) and MRS	✓		
Retrieve investigational products		✓	✓
Retrieve questionnaires (SF-36) and MRS		✓	✓
Use of investigational product check		✓	✓
Recording reactions (AE, SAE)		✓	✓

Supplementary Figure S1. Study flow and schedule of assessments chart

Supplementary Table S2. SF-36 QoL questionnaire.

Today's date: ___/___/_____ Vol. ID ___________

First name: ______________________________ Surname: ______________________________

SF-36 Questionnaire

For ALL questions, please tick, cross or colour the circle that most closely matches your response. There are no right or wrong answers. Please answer ALL questions.

**1. In general, would you say your health is:**	Poor ○	Fair ○	Good ○	Very good ○	Excellent ○
**2. Compared to one year ago, how would you rate your health general in now?**	Much worse now than one year ago ○	Somewhat worse than one year ago ○	About the same as one year ago ○	Somewhat better than one year ago ○	Much better than one year ago ○

The following questions are about activities you might do during a typical day. Does your health now limit you in these activities? If so, how much?

	No, not limited at all	Yes, limited a little	Yes, limited a lot
**3. Vigorous activities, such as running, lifting heavy objects, participating in strenuous sports**	○	○	○
**4. Moderate activities, such as moving a table pushing a vacuum cleaner, bowling, or playing**	○	○	○
**5. Lifting or carrying groceries**	○	○	○
**6. Climbing several flights of stairs**	○	○	○
**7. Climbing one flight of stairs**	○	○	○
**8. Bending, kneeling or stooping**	○	○	○
**9. Walking more than a mile**	○	○	○
**10. Walking several blocks**	○	○	○
**11. Walking one block**	○	○	○
**12. Bathing or dressing yourself**	○	○	○

During the past 4 weeks, how much of the time have you had any of the following problems with your work or other daily activities as a result of your physical health?

	None of the time	A little of the time	Some of the time	Most of the time	All of the time
**13. Cut down on the amount of time you spent on work or other activities**	○	○	○	○	○
**14. Accomplished less than you would like**	○	○	○	○	○
**15. Were limited in the kind of work or other activities**	○	○	○	○	○
**16. Had difficulty performing the work or other activities (e.g., it took extra effort)**	○	○	○	○	○

During the past 4 weeks, how much of the time have you had any of the following problems with your work or other regular daily activities as a result of any emotional problems (such as feeling depressed or anxious)?

	None of the time	A little of the time	Some of the time	Most of the time	All of the time
**17. Cut down on the amount of time you spent on work or other activities**	○	○	○	○	○
**18. Accomplished less than you would like**	○	○	○	○	○
**19. Did work or other activities less carefully than usual**	○	○	○	○	○
**20. During the past 4 weeks, to what extent has your physical health or emotional problems interfered with your normal social activities with family, friends, neighbors, or groups?**	Not at all ○	Slightly ○	Moderately ○	Quite a bit ○	All of the time ○

---> continue on next page

---> to be continued

**21. How much bodily pain have you had during the past 4 weeks?**	None<br>○	Very mild<br>○	Mild<br>○	Moderate<br>○	Severe<br>○	Very severe<br>○

**22. During the past 4 weeks, how much did pain interfere with your normal work (including both work outside the home and housework)?**	Not at all<br>○	A little bit<br>○	Moderately<br>○	Quite a bit<br>○	Extremely<br>○

These questions are about how you feel and how things have been with you during the past 4 weeks. For each question, please give the one answer that comes closest to the way you have been feeling. How much of the time during the past 4 weeks...

	None of the time	A little of the time	Some of the time	Most of the time	All of the time
**23. did you feel full of life?**	○	○	○	○	○
**24. have you been very nervous?**	○	○	○	○	○
**25. have you felt so down in the dumps that nothing could cheer you up?**	○	○	○	○	○
**26. have you felt calm and peaceful?**	○	○	○	○	○
**27. did you have a lot of energy?**	○	○	○	○	○
**28. have you felt downhearted and depressed?**	○	○	○	○	○
**29. did you feel worn out?**	○	○	○	○	○
**30. have you been happy?**	○	○	○	○	○
**31. did you feel tired?**	○	○	○	○	○

**32. During the past 4 weeks, how much of the time has your physical health or emotional problems interfered with your social activities (like visiting friends, relatives, etc.)?**	None of the time<br>○	A little of the time<br>○	Some of the time<br>○	Most of the time<br>○	All of the time<br>○

How TRUE or FALSE is each of the following statements for you?

	Definitely false	Mostly false	Don’t know	Mostly true	Definitely true
**33. I seem to get sick a little easier than other people**	○	○	○	○	○
**34. I am as healthy as anybody I know**	○	○	○	○	○
**35. I expect my health to get worse**	○	○	○	○	○
**36. My health is excellent**	○	○	○	○	○

Supplementary Table S3. Menopause Rating Scale (MRS).

Today's date: ___/___/_____ Vol. ID ___________

First name: ____________________________ Surname: ____________________________

Menopause Rating Scale (MRS) Questionnaire

Which of the following symptoms apply to you at this time? (X ONE Box For EACH Symptom). For Symptoms That Do Not Apply, Please Mark "None").

	None	Mild	Moderate	Severe	Extremely severe
**Score=**	**0**	**1**	**2**	**3**	**4**
1. Hot flashes, sweating (episodes of sweating)	○	○	○	○	○
2. Heart discomfort (unusual awareness of heart beat, heart skipping, heart racing, tightness)	○	○	○	○	○
3. Sleep problems (difficulty in falling asleep, difficulty in sleeping through the night, waking up early)	○	○	○	○	○
4. Depressive mood (feeling down, sad, on the verge of tears, lack of drive, mood swings)	○	○	○	○	○
5. Irritability (feeling nervous, inner tension, feeling aggressive)	○	○	○	○	○
6. Anxiety (inner restlessness, feeling panicky)	○	○	○	○	○
7. Physical and mental exhaustion (general decrease in performance, impaired memory, decrease in concentration, forgetfulness)	○	○	○	○	○
8. Sexual problems (change in sexual desire, in sexual activity and satisfaction)	○	○	○	○	○
9. Bladder problems (difficulty in urinating, increased need to urinate, bladder incontinence)	○	○	○	○	○
10. Dryness of vagina (sensation of dryness or burning in the vagina, difficulty with sexual intercourse)	○	○	○	○	○
11. Joint and muscular discomfort (pain in the joints, rheumatoid complaints)	○	○	○	○	○

Supplementary Table S4. Subjects' baseline demographic and clinical characteristics.

	Active	Placebo	Units
**Sex**			
**Male, % (n)**	50 (15)	50 (15)	% (no.)
**Female, % (n)**	50 (15)	50 (15)	% (no.)
**GSH**	949.4±63.9	897.4±57.1	μM
**GSSG**	362.4±16.7	350.6±16.4	μM
**GSH/GSSG**	2.7±0.2	2.7±0.2	Ratio
**d-ROMS**	392.3±15.5	398.6±13.1	U.CARR
**Serum TNF-α levels**	7.19±0.07	7.32±0.10	pg/mL
**Complete blood cell count**			
WBC	6.6±0.3	6.6±0.3	$10^3/\mu L$
RBC	4.8±0.1	4.9±0.1	$10^6/\mu L$
Hb	14.4±0.2	14.0±0.3	g/dl
Hct	42.0±0.6	41.2±0.8	%
PLT	234.4±9.5	223.7±12.2	$10^3/\mu L$
**Biochemistry test**			
BUN	35.6±1.5	38.6±1.4	mg/dl
Cholesterol	207.0±9.0	208.7±6.2	mg/dl
HDL-C	76.7±4.6	66.2±2.7	mg/dl
LDL-C	114.2±8.0	130.8±6.4	mg/dl
Triglycerides	109.7±7.6	105.5±5.7	mg/dl
Albumin	4.6±0.1	4.6±0.0	g/dl
Total bilirubin	0.7±0.1	0.6±0.1	mg/dl
ALP	73.1±3.8	75.3±4.2	U/L
r-GT	23.5±2.8	20.7±2.1	U/L
Creatinine	0.85±0.03	0.81±0.04	mg/dl
hs-CRP	2.1±0.3	1.9±0.3	mg/l
**Urinalysis**			
Specific Gravity	1.02±0.00	1.02±0.00	g/L
pH	5.6±0.1	5.6±0.1	---
WBC	17.0±5.2	20.2±7.0	μL
Erythrocytes	9.0±2.1	13.8±2.9	μL
Nitrite	Absent	Absent	---
Protein	Absent	Absent	---
Glucose	Absent	Absent	---
Ketone body	Absent	Absent	---
Urobilinogen	Absent	Absent	---
Bilirubin	Absent	Absent	---

Data are average (± standard error) or number of subjects (n or %). Legend. WBC White Blood Cells; RBC Red Blood cells; Hb Hemoglobin; Hct Hematocrit; PLT Platelet count; BUN Blood Urea Nitrogen, Azotemia; HDL-C High-Density Lipoprotein Cholesterol; LDL-C Low-Density Lipoprotein Cholesterol; ALP Alkaline Phosphatase; r-GT Gamma-glutamyl Transferase; hs-CRP High-sensitivity C-reactive Protein; WBC White Blood Cells, Leukocytes; Occult blood erythrocytes.

Supplementary Table S5. Blood and Urine analysis in the **active** treatment arm. WBC White Blood Cells; RBC Red Blood cells; Hb Hemoglobin; Hct Hematocrit; PLT Platelet count; BUN Blood Urea Nitrogen, azotemia; HDL-C High-Density Lipoprotein Cholesterol; LDL-C Low-Density Lipoprotein Cholesterol; ALP Alkaline Phosphatase; r-GT Gamma-glutamyl Transferase: hs-CRP High-sensitivity C-reactive Protein; WBC White Blood Cells, leukocytes.

	Parameter	T0	T8	Normality range	units
Complete blood cell count (n=29*)	WBC	6.6±0.3	6.7±0.3	4.00 - 9.0	10^3/μL
	RBC	4.8±0.1	4.8±0.1	3.80 - 5.30	10^6/μL
	Hb	14.4±0.2	14.3±0.2	12.0 - 18.0	g/dl
	Hct	42.0±0.6	42.1±0.7	36.0 - 56.0	%
	PLT	234.4±9.5	242.3±8.3	120.0 - 380.0	10^3/μL
Biochemistry test (n=29*)	BUN	35.6±1.5	32.6±1.4	10 - 50	mg/dl
	Cholesterol	207.0±9.0	204.5±7.0	140 - 200	mg/dl
	HDL-C	76.7±4.6	81.6±5.7	> 45	mg/dl
	LDL-C	114.2±8.0	114.2±5.9	0 - 135	mg/dl
	Triglycerides	109.7±7.6	105.8±8.1	0 - 200	mg/dl
	Albumin	4.6±0.1	4.6±0.1	3.5 - 5.5	g/dl
	Total bilirubin	0.7±0.1	0.6±0.1	0.0 - 1.20	mg/dl
	ALP	73.1±3.8	76.4±4.5	45 - 98	U/L
	r-GT	23.5±2.8	23.5±2.7	5 - 36	U/L
	Creatinine	0.85±0.03	0.83±0.03	0.50 - 0.90	mg/dl
	hs-CRP	2.1±0.3	2.4±0.4	0.0 - 5.00	mg/l
Urinalysis (n=29*)	Specific Gravity	1.02±0.00	1.02±0.00	1.01 - 1.025	g/L
	pH	5.6±0.1	5.7±0.1	5.0 - 7.5	---
	WBC	17.0±5.2	17.0±7.0	0 - 25	mL
	Erythrocytes	9.0±2.1	15.6±5.9	0 - 30	mL
	Nitrite	Absent	Absent	Absent	---
	Protein	Absent	Absent	Absent	---
	Glucose	Absent	Absent	Absent	mg/dl
	Ketone body	Absent	Absent	Absent	---
	Urobilinogen	Absent	Absent	Absent	---
	Bilirubin	Absent	Absent	Absent	---

* The basal analysis of subject no. 26 (in the active treatment arm) was lost.

Supplementary Table. S6 Blood and Urine analysis in the **placebo** treatment arm. WBC White Blood Cells; RBC Red Blood cells; Hb Hemoglobin; Hct Hematocrit; PLT Platelet count; BUN Blood Urea Nitrogen, azotemia; HDL-C High-Density Lipoprotein Cholesterol; LDL-C Low-Density Lipoprotein Cholesterol; ALP Alkaline Phosphatase; r-GT Gamma-glutamyl Transferase: hs-CRP High-sensitivity C-reactive Protein; WBC White Blood Cells, leukocytes.

	Parameter	T0	T8	Normality range	units
Complete blood cell count (n=30)	WBC	6.6±0.3	6.6±0.3	4.00 - 9.0	10^3/μL
	RBC	4.9±0.1	5.0±0.1	3.80 - 5.30	10^6/μL
	Hb	14.0±0.3	14.0±0.3	12.0 - 18.0	g/dl
	Hct	41.2±0.8	41.6±0.8	36.0 - 56.0	%
	PLT	223.7±12.2	227.1±11.5	120.0 - 380.0	10^3/μL
Biochemistry test (n=30)	BUN	38.6±1.4	34.2±1.6	10 - 50	mg/dl
	Cholesterol	208.7±6.2	204.4±4.4	140 - 200	mg/dl
	HDL-C	66.2±2.7	72.6±2.8	> 45	mg/dl
	LDL-C	130.8±6.4	115.3±5.0	0 - 135	mg/dl
	Triglycerides	105.5±5.7	114.2±5.3	0 - 200	mg/dl
	Albumin	4.6±0.0	4.5±0.1	3.5 - 5.5	g/dl
	Total bilirubin	0.6±0.1	0.6±0.1	0.0 - 1.20	mg/dl
	ALP	75.3±4.2	77.6±3.6	45 - 98	U/L
	r-GT	20.7±2.1	21.1±2.1	5 - 36	U/L
	Creatinine	0.81±0.04	0.78±0.03	0.50 - 0.90	mg/dl
	hs-CRP	1.9±0.3	2.2±0.3	0.0 - 5.00	mg/l
Urinalysis (n=30)	Specific Gravity	1.02±0.00	1.02±0.00	1.01 - 1.025	g/L
	pH	5.6±0.1	5.7±0.1	5.0 - 7.5	---
	WBC	20.2±7.0	18.8±8.5	0 - 25	mL
	Erythrocytes	13.8±2.9	9.4±2.1	0 - 30	mL
	Nitrite	Absent	Absent	Absent	---
	Protein	Absent	Absent	Absent	---
	Glucose	Absent	Absent	Absent	mg/dl
	Ketone body	Absent	Absent	Absent	---
	Urobilinogen	Absent	Absent	Absent	---
	Bilirubin	Absent	Absent	Absent	---
